# Supplementary material for: Changes in SARS-CoV-2 seroprevalence and population immunity in Finland, 2020–2022
Source: Emerg Microbes Infect. 2023 Jun 25;12(2):2222849. doi: 10.1080/22221751.2023.2222849 (PMC10291939; doi:10.1080/22221751.2023.2222849)
Supplement: Supplemental Material [file TEMI_A_2222849_SM7407.docx]

# Supplementary material

## Population surveys

The size of population surveys was adapted to the expected true seroprevalence in the population to achieve a predefined 2–3 percentage point accuracy in the 90% interval estimate of seroprevalence. Due to the properties of the binomial distribution, the required sample size is highest when the true seroprevalence is close to 50%, and lowest when it is close to 0% or 100%. Initially, the target size of each survey was 750, which is sufficient for 2 percentage point accuracy in the estimate in a population where seroprevalence is low (<10%). As seroprevalence estimates remained below 5%, the size of the surveys was lowered. As the seroprevalence was expected to have increased by 2022 due to the emergence of more infectious variants, the size of the sample was increased accordingly, with a target size of 1300 per survey. This latter sample size is sufficient for 2 percentage point accuracy when the prevalence is below 20%, and 3 percentage point accuracy when the prevalence is 50%. Note that in the current study, we report 95% confidence intervals for seroprevalence. The Helsinki and Uusimaa healthcare district (HUS) had the biggest weight in surveys (53% of all invitations) due to its larger population and it being the area where the outbreak first started in Finland, subsequently progressing to other areas.

## FMIA assay specifications and thresholds for positivity

Assay specificity and sensitivity were determined by analysing sera (n=402) collected in mid-2019 (negative controls) and sera collected in 2020 from subjects (n=87) with PCR-confirmed COVID-19 14-51 days after symptom onset and no COVID-19 vaccination history (positive controls). The positive control sample material has been previously described[1]. Samples collected from April to September 2020 (n=3954) were only analysed for N-IgG, and the samples thereafter were analysed for N-IgG and S-IgG (n=5840).

Samples collected from April 2020 to December 2021 (n=7039) were analysed as duplicates diluted 1:100. The threshold for N-IgG positivity during this period was >6.7 BAU/ml resulting in 100% specificity and 95.8% sensitivity. Samples with >0.9 BAU/ml IgG to receptor binding domain and >1.6 BAU/ml IgG to full-length spike glycoprotein were considered S-IgG positive and the assay specificity and sensitivity were 100%.

Samples collected in 2022 (n=2755) were analysed as duplicates diluted 1:100 and 1:1600. The threshold for N-IgG positivity during this period was >7 BAU/ml resulting in 92.8% specificity and 100% sensitivity. Samples with >3 BAU/ml IgG to receptor binding domain and >2 BAU/ml IgG to full-length spike glycoprotein were considered S-IgG positive, and the assay specificity and sensitivity were 100%.

## Supplementary tables and figures

**Table S1. Description of the random population surveys**

| **Survey** | **Week/year** | **Sample size**  **total/per district** | **Invited subjects** | **Participated subjects** | **Participation rate** | **Healthcare district** |
| --- | --- | --- | --- | --- | --- | --- |
| 1 | 14/2020 | 750 | 733 | 468 | 64% | HUS |
| 2 | 15/2020 | 750 | 728 | 451 | 62% | HUS |
| 3 | 16/2020 | 1000/200 | 989 | 509 | 51% | All five |
| 4 | 17/2020 | 1000/200 | 980 | 469 | 48% | All five |
| 5 | 17/2020 | 1000/200 | 980 | 480 | 49% | All five |
| 6 | 18/2020 | 750/150 | 738 | 313 | 42% | All five |
| 7 | 19/2020 | 400/80 | 392 | 162 | 41% | All five |
| 8 | 21/2020 | 400/80 | 394 | 145 | 37% | All five |
| 9 | 22/2020 | 400/80 | 398 | 171 | 43% | All five |
| 10 | 22/2020 | 900/500^§^/80 | 876 | 314 | 36% | All five |
| 11 | 25/2020 | 400/80 | 391 | 132 | 34% | All five |
| 12 | 29/2020 | 400/80 | 391 | 130 | 33% | All five |
| 13 | 31/2020 | 400/80 | 396 | 138 | 35% | All five |
| 14 | 33/2020 | 400^*^ | 392 | 113 | 29% | All five |
| 15 | 35/2020 | 400^*^ | 390 | 113 | 29% | All five |
| 16 | 37/2020 | 400^*^ | 392 | 112 | 29% | All five |
| 17 | 39/2020 | 400^*^ | 387 | 113 | 29% | All five |
| 18 | 41/2020 | 400^*^ | 392 | 107 | 27% | All five |
| 19 | 43/2020 | 400^*^ | 392 | 104 | 27% | All five |
| 20 | 45/2020 | 600^*#^ | 599 | 174 | 29% | All five |
| 21 | 47/2020 | 600^*#^ | 600 | 170 | 28% | All five |
| 22 | 49/2020 | 600^*#^ | 601 | 171 | 28% | All five |
| 23 | 52/2020 | 600^*#^ | 594 | 164 | 28% | All five |
| 24 | 02/2021 | 600^*#^ | 595 | 160 | 27% | All five |
| 25 | 05/2021 | 600^*#^ | 597 | 192 | 32% | All five |
| 26 | 07/2021 | 600^*#^ | 588 | 178 | 30% | All five |
| 27 | 09/2021 | 600^*#^ | 594 | 184 | 31% | All five |
| 28 | 11/2021 | 400 | 382 | 111 | 29% | HUS |
| 29 | 14/2021 | 600^*#^ | 599 | 181 | 30% | All five |
| 30 | 15/2021 | 400 | 391 | 119 | 30% | HUS |
| 31 | 17/2021 | 600^*#^ | 601 | 147 | 24% | All five |
| 32 | 19/2021 | 400 | 390 | 101 | 26% | HUS |
| 33 | 21/2021 | 600^*#^ | 603 | 123 | 20% | All five |
| 34 | 23/2021 | 400 | 385 | 83 | 22% | HUS |
| 35 | 27/2021 | 400 | 386 | 86 | 22% | HUS |
| 36 | 41/2021 | 750 | 728 | 153 | 21% | HUS |
| 37 | 10/2022 | 2500 | 2426 | 377 | 16% | HUS |
| 38 | 17/2022 | 2500 | 2424 | 458 | 19% | HUS |
| 39 | 19/2022 | 4000/1000 | 3929 | 731 | 19% | All excl. HUS |
| 40 | 40/2022 | 6500/2500^§^/1000 | 6358 | 1190 | 19% | All five |

HUS= Helsinki and Uusimaa healthcare district

All five = HUS, Pirkanmaa, Northern Ostrobothnia, Northern Savonia and Southwest Finland

*sample size per district adjusted by population

# sample size for HUS district doubled

§ sample size for HUS district

**Table S2. The number of participants per year and month.**

|  |  | **2020** | **2021** | **2022** |
| --- | --- | --- | --- | --- |
| Q1 | January | 0 | 53 | 1 |
|  | February | 0 | 413 | 0 |
|  | March | 0 | 418 | 168 |
| Q2 | April | 1460 | 314 | 87 |
|  | May | 1305 | 319 | 456 |
|  | June | 685 | 215 | 791 |
| Q3 | July | 201 | 43 | 38 |
|  | August | 244 | 83 | 14 |
|  | September | 242 | 3 | 8 |
| Q4 | October | 245 | 13 | 258 |
|  | November | 261 | 132 | 879 |
|  | December | 382 | 10 | 53 |

**Table S3. Study population compared to Uusimaa region and Finland’s 18-85-year-old population structure at the end of 2021.**

|  |  | **Study population**  **n=9794** | **Uusimaa, Finland,**  **18-85-year-olds**  **(n=1.35 million)** | **Whole Finland,  18-85-year-olds**  **(n=4.36 million)** |
| --- | --- | --- | --- | --- |
| Age | 18-29 | 11% | 19% | 18 % |
|  | 30-44 | 25% | 29% | 25 % |
|  | 45-64 | 44% | 32% | 32 % |
|  | 65-85 | 19%^a^ | 21% | 26 % |
| Sex | Female | 61% | 51% | 50 % |
|  | Male | 39% | 49% | 50 % |
| Native language | Finnish or Swedish | 97% | 85% | 91 % |
|  | Other | 3% | 15% | 9 % |
| Reside in Uusimaa | | 54% | 100% | 31% |
| Vaccinated for COVID-19^b^ | | 96%^d^ | 88% | 88% |
| Registered COVID-19 cases^c^ | | 2518 (26%)^e^ | 457 528 (34%) | 1 219 008 (28%) |

^a^ 65-70-year-olds in 2021-2021 and 65-85-year-olds in 2022.

^b^ Have received at least one COVID-19 vaccine dose before 15^th^ of December 2021, i.e. would have developed vaccine-mediated immunity by 2022.

^c^ January 1^st^ 2020 to December 31^st^ 2022.

^d^ Vaccinated before or after sample collection.

^e^ Case registered before or after sample collection.

**Table S4. The number of serological samples collected in Finland’s different healthcare districts per year quartile.**

|  |  | **Number of participants per healthcare district, % of the quartile’s samples** | | | | |
| --- | --- | --- | --- | --- | --- | --- |
| Year | Quartile | Helsinki and Uusimaa | Pirkanmaa | Northern Ostrobothnia | Northern Savonia | Southwest Finland |
| 2020 | Q2, n=3450 | 46% (n=1603) | 15% (n=517) | 12% (n=420) | 14% (n=481) | 12% (n=429) |
|  | Q3, n=687 | 35% (n=242) | 20% (n=135) | 16% (n=107) | 13% (n=88) | 17% (n=115) |
|  | Q4, n=888 | 65% (n=580) | 13% (n=114) | 7% (n=63) | 7% (n=62) | 8% (n=69) |
| 2021 | Q1, n=884 | 70% (n=618) | 11% (n=101) | 7% (n=62) | 4% (n=37) | 7% (n=66) |
|  | Q2, n=847 | 83% (n=705) | 6% (n=50) | 5% (n=44) | 3% (n=22) | 3% (n=26) |
|  | Q3, n=129 | 96% (n=124) | 3% (n=4) | 1% (n=1) | 0% (n=0) | 0% (n=0) |
|  | Q4, n=155 | 100% (n=155) | 0% (n=0) | 0% (n=0) | 0% (n=0) | 0% (n=0) |
| 2022 | Q1, n=169 | 100% (n=169) | 0% (n=0) | 0% (n=0) | 0% (n=0) | 0% (n=0) |
|  | Q2, n=1335 | 48% (n=645) | 13% (n=179) | 11% (n=152) | 14% (n=193) | 12% (n=166) |
|  | Q3, n=60 | 33% (n=20) | 20% (n=12) | 5% (n=3) | 15% (n=9) | 27% (n=16) |
|  | Q4, n=1190 | 38% (n=451) | 16% (n=191) | 16% (n=188) | 17% (n=202) | 13% (n=158) |
| Total, n=9794 | | 54% (n=5312) | 13% (n=1303) | 11% (n=1040) | 11% (n=1094) | 11% (n=1045) |

**Table S5. SARS-CoV-2 nucleoprotein (N-IgG) and spike glycoprotein (S-IgG) seropositivity by year quartile and age group.**

| **Year** | **Quartile** | **Analysed samples, n** | **Age** | **N-IgG positives %, (n)** | **S-IgG positives %, (n)** |
| --- | --- | --- | --- | --- | --- |
| 2020 | Q2 | N-IgG:3450  S-IgG:0 | 18-29 | 0.8% (4/523) | NA (0/0) |
|  |  |  | 30-44 | 2.3% (23/1005) | NA (0/0) |
|  |  |  | 45-64 | 2.2% (35/1562) | NA (0/0) |
|  |  |  | 65-70 | 3.1% (11/360) | NA (0/0) |
|  | Q3 | N-IgG:687  S-IgG:183 | 18-29 | 4.5% (3/66) | 0.0% (0/10) |
|  |  |  | 30-44 | 2.9% (6/204) | 4.8% (3/62) |
|  |  |  | 45-64 | 2.2% (7/317) | 2.3% (2/88) |
|  |  |  | 65-70 | 5.0% (5/100) | 0.0% (0/23) |
|  | Q4 | N-IgG:888  S-IgG:888 | 18-29 | 6.0% (7/117) | 3.4% (4/117) |
|  |  |  | 30-44 | 2.2% (6/269) | 0.7% (2/269) |
|  |  |  | 45-64 | 2.8% (11/390) | 1.5% (6/390) |
|  |  |  | 65-70 | 0.0% (0/112) | 0.0% (0/112) |
| 2021 | Q1 | N-IgG:884  S-IgG:884 | 18-29 | 2.7% (3/112) | 4.5% (5/112) |
|  |  |  | 30-44 | 2.4% (6/249) | 5.2% (13/249) |
|  |  |  | 45-64 | 3.6% (15/419) | 6.9% (29/419) |
|  |  |  | 65-70 | 1.9% (2/104) | 4.8% (5/104) |
|  | Q2 | N-IgG:847  S-IgG:847 | 18-29 | 4.7% (6/127) | 13.4% (17/127) |
|  |  |  | 30-44 | 7.3% (17/232) | 27.6% (64/232) |
|  |  |  | 45-64 | 4.8% (19/399) | 51.1% (204/399) |
|  |  |  | 65-70 | 3.4% (3/89) | 74.2% (66/89) |
|  | Q3 | N-IgG:129  S-IgG:129 | 18-29 | 0.0% (0/10) | 70.0% (7/10) |
|  |  |  | 30-44 | 6.3% (3/48) | 77.1% (37/48) |
|  |  |  | 45-64 | 3.6% (2/55) | 90.9% (50/55) |
|  |  |  | 65-70 | 0.0% (0/16) | 93.8% (15/16) |
|  | Q4 | N-IgG:155  S-IgG:155 | 18-29 | 7.1% (1/14) | 92.9% (13/14) |
|  |  |  | 30-44 | 0.0% (0/41) | 82.9% (34/41) |
|  |  |  | 45-64 | 12.8% (10/78) | 89.7% (70/78) |
|  |  |  | 65-70 | 4.5% (1/22) | 100.0% (22/22) |
| 2022 | Q1 | N-IgG:169  S-IgG:169 | 18-29 | 33.3% (3/9) | 100.0% (9/9) |
|  |  |  | 30-44 | 41.2% (14/34) | 100.0% (34/34) |
|  |  |  | 45-64 | 30.4% (21/69) | 100.0% (69/69) |
|  |  |  | 65-85 | 19.3% (11/57) | 100.0% (57/57) |
|  | Q2 | N-IgG:1335  S-IgG:1335 | 18-29 | 55.4% (41/74) | 95.9% (71/74) |
|  |  |  | 30-44 | 44.9% (96/214) | 95.3% (204/214) |
|  |  |  | 45-64 | 35.6% (195/548) | 98.2% (538/548) |
|  |  |  | 65-85 | 27.9% (139/499) | 99.6% (497/499) |
|  | Q3 | N-IgG:60  S-IgG:60 | 18-29 | 100.0% (4/4) | 100.0% (4/4) |
|  |  |  | 30-44 | 64.3% (9/14) | 92.9% (13/14) |
|  |  |  | 45-64 | 38.7% (12/31) | 100.0% (31/31) |
|  |  |  | 65-85 | 36.4% (4/11) | 100.0% (11/11) |
|  | Q4 | N-IgG:1190  S-IgG:1190 | 18-29 | 71.2% (37/52) | 98.1% (51/52) |
|  |  |  | 30-44 | 55.8% (96/172) | 97.7% (168/172) |
|  |  |  | 45-64 | 53.6% (241/450) | 98.9% (445/450) |
|  |  |  | 65-85 | 39.7% (205/516) | 99.2% (512/516) |

Table S6. Key developments in Finland’s national COVID-19 vaccination programme from 2020 to 2022 and simplified timeline of available vaccine products in Finland, adapted from [2] and [3].

| **Year** | **Key timepoints in Finland’s COVID-19 vaccination programme** | **Vaccines used** |
| --- | --- | --- |
| 2020 | **December**: First COVID-19 vaccinations begin in healthcare workers caring for COVID-19 patients. |  |
| 2021 | **January**: Vaccinations in nursing homes begin.  **February**: Vaccinations in risk groups begin and are organized stepwise, with those at highest risk[2] being offered the vaccine first.  **April**: Vaccinations of non-risk groups begin and are offered stepwise to the population starting from 60–69-year-olds, followed by 50–59, 40–49, 30–39, 16–29 and 12–15-year-olds.  **August**: Vaccines available for all aged 12 years and older. Vaccines recommended also for pregnant women.  **September**: Only one dose recommended to those with previous PCR-confirmed COVID-19 infection. Severely immunocompromised eligible for 3^rd^ vaccine dose.  **October**: Recommendation for 3^rd^ dose for risk groups.  **November**: Recommendation for 3^rd^ dose for healthcare workers caring for risk groups.  **December**: Recommendation for 3^rd^ dose for all over 18-year-olds and 4^th^ doses recommended for the severely immunocompromised. 5–11-year-olds become eligible for vaccinations. | Comirnaty  Spikevax  Vaxzevria  Janssen  Nuvaxovid  Variant^b^  Variant^a^ |
| 2022 | **March**: 3^rd^ dose recommended to 12–17-year-olds belonging to risk groups. 4^th^ dose recommended to over 80-year-olds and those living in assisted care units.  **June**: 4^th^ doses recommended to 70–79-year-olds belonging to risk groups.  5^th^ dose recommended to the severely immunocompromised.  **September**: COVID-19 variant (BA.1 and BA.4/5) vaccines available to use as booster doses.  **December**: BA.4/5 vaccine available to use in primary vaccination series. |  |

^a^ Comirnaty BA.1 and BA.4/5 vaccines
^b^ Spikevax BA.1 vaccine.


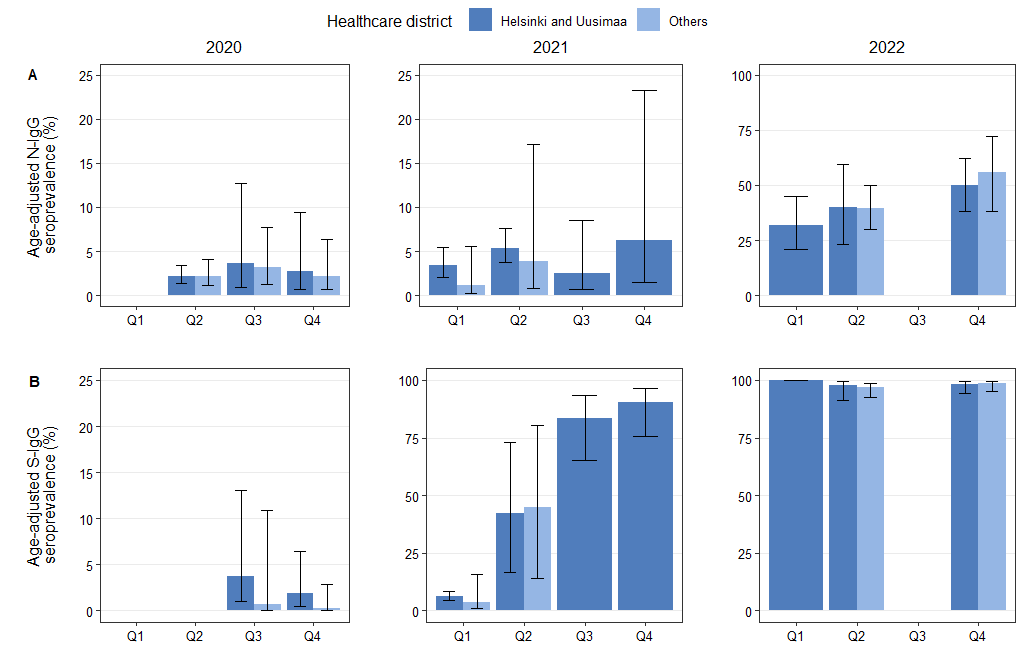
**Figure S1.** **SARS-CoV-2 seroprevalence in Finnish adults from April 2020 to December 2022 by healthcare district and quartiles (Q1-Q4).** Participants from Pirkanmaa, Northern Ostrobothnia, Northern Savonia and Southwest Finland healthcare districts are summarised in “Others”. In 2020-2021 the oldest age group comprised of 65-70-year-olds and was extended to 65-85-year-olds in 2022. Error bars represent 95% confidence intervals. Time points with less than 30 subjects are excluded and the y-axes have different scales within rows. **A.** Age-adjusted SARS-CoV-2 nucleoprotein (N-IgG) seroprevalence. **B.** Age-adjusted SARS-CoV-2 spike glycoprotein (S-IgG) seroprevalence.

## Supplementary references

[1] Dub T, Solastie A, Hagberg L, et al. High secondary attack rate and persistence of SARS-CoV-2 antibodies in household transmission study participants, Finland 2020-2021. Front Med. 2022;9:876532.

[2] Finnish Institute for Health and Welfare. Arranging COVID-19 vaccinations in Finland - THL [Internet]. 2022 [cited 2023 Mar 1]. Available from: https://thl.fi/en/web/infectious-diseases-and-vaccinations/what-s-new/coronavirus-covid-19-latest-updates/vaccines-and-coronavirus/arranging-covid-19-vaccinations-in-finland.

[3] Koronarokotusten järjestäminen Suomessa - THL [Internet]. Terveyden Ja Hyvinvoinnin Laitos. [cited 2023 May 10]. Available from: https://thl.fi/fi/web/infektiotaudit-ja-rokotukset/ajankohtaista/ajankohtaista-koronaviruksesta-covid-19/rokotteet-ja-koronavirus/koronarokotusten-jarjestaminen-suomessa.
